# The Relationship Between the Recognition of Basic Emotions and Negative Symptoms in Individuals With Schizophrenia Spectrum Disorders – An Exploratory Study

**DOI:** 10.3389/fpsyt.2022.865226

**Published:** 2022-04-27

**Authors:** Marco Zierhut, Kerem Böge, Niklas Bergmann, Inge Hahne, Alice Braun, Julia Kraft, Thi Minh Tam Ta, Stephan Ripke, Malek Bajbouj, Eric Hahn

**Affiliations:** ^1^Department of Psychiatry and Psychotherapy, Charité – Universitätsmedizin Berlin, Berlin, Germany; ^2^BIH Charitè Junior Clinician Scientist Program, BIH Biomedical Innovation Academy, Berlin Institute of Health at Charité – Universitätsmedizin Berlin, Berlin, Germany; ^3^Department of Psychiatry and Psychotherapy, Charité – Universitätsmedizin Berlin, Berlin, Germany

**Keywords:** schizophrenia spectrum disorders, negative symptoms, emotion recognition, schizophrenia, psychosis

## Abstract

Current research suggests that emotion recognition is impaired in individuals affected by schizophrenia spectrum disorders (SSD). However, the specific impact of negative symptoms on the ability to recognize single basic emotions has not yet been explored sufficiently and is the aim of the present study. A sample of *N* = 66 individuals diagnosed with SSD was recruited at the Charité – Universitätsmedizin Berlin. In a first step, correlation analyses were conducted between seven different negative symptom subdomains of the Positive and Negative Syndrome Scale (PANSS) and the accuracy and latency in recognizing the six basic emotions (anger, disgust, fear, happiness, sadness, surprise) using the Emotion Recognition Task (ERT) of the Cambridge Neuropsychological Test Automated Battery (CANTAB). The significant correlations were subjected to linear regression models that controlled for the significant covariates diagnoses, age, sex, and education. Results revealed that in individuals with SSD the negative symptom domain of blunted affect significantly predicted the accuracy of emotion recognition performance (*p* < 0.05), particularly, when recognizing happiness (*p* < 0.05). Additionally, we found that stereotyped thinking also predicted the performance of emotion recognition, especially the response latency (*p* < 0.05) and difficulty in abstract thinking predicted the recognition of fear (*p* < 0.05). However, the nominal significances did not withstand correction for multiple tests and therefore need to be followed up in further studies with a larger sample.

## Introduction

Social cognition is a crucial domain for interacting with other people and participation in society. It has been defined as the psychological processes that underlie social interactions, including perception, experience sharing, mentalizing, and experiencing and regulating emotion and information about other people and about ourselves (1, 2). Among this heterogeneous category of processes, the most important domains of social cognition include the perception of social cues like emotion perception (1–4). Individuals affected by schizophrenia spectrum disorders (SSD) show significant impairments in their ability to recognize and express emotions, as already described by Kraepelin (2, 5, 6). Previously, studies of emotion deficits in schizophrenia focused on three main domains: expression, experience, and recognition of emotions. A review of 110 studies revealed marked impairment in all three domains in individuals with SSD (7). It is essential to differentiate between emotion recognition – which refers to the ability to recognize an expression of emotion such as disgust - and finding a face disgusting, which is called subjective appraisal (7). Emotion recognition has been linked to emotional intelligence and social perception, whereas appraisal has been linked to emotion processing (7). Gur et al. (8) reported an association of greater impairment for individuals with SSD in emotion recognition via facial stimuli with the severity of negative symptoms. Social cognitive impairments and negative symptoms are core features of SSD closely associated with high burden due to impairments in social functioning and quality of life already apparent prior to illness onset (2, 5, 6, 9–12). For example attenuated impairment in emotion recognition manifests in unaffected first-degree relatives, ultra-high-risk individuals (13, 14), and young individuals with first-episode schizophrenia (5, 15, 16). A study found that young individuals with schizophrenia were impaired in their ability to recognize faces and gestures from movie scenes during the first two to 3 years of their illness after symptom onset (15, 17). According to Comparelli, Corigliano (13), facial emotion recognition impairment already present in ultra-high-risk individuals remained stable across the course of illness. Deficits in emotion recognition may be one of the most consistent and severe aspects of interpersonal problems (15) and impairment of social skills (18) and are related to poorer social outcomes in schizophrenia (19). Some researchers argue emotion recognition deficits are long-term stable features of schizophrenia (20). In one model, negative symptoms, emotion recognition latency, and processing speed were significant predictors of social functioning in individuals at ultra-high risk for psychosis (21, 22). Another model for schizophrenia onset included face emotion processing and negative symptoms as predictors of transition in individuals at high clinical risk to schizophrenia with an accuracy of 96% (11). Accordingly, this represents a particularly relevant field of research since both negative symptoms and impairments in social cognition could be apparent prior to illness onset with psychotic symptoms (10, 11).

Impaired emotional functioning has been linked to negative symptoms and can be conceptualized as a fundamental clinical feature in SSD (7, 23, 24). Newer discussions on deficits in emotion recognition raise the question of whether those belong to the negative symptom spectrum or form a separate domain in SSD (1, 25, 26). According to recent models, negative symptoms are also described as deficient social cognitions (27). The existing data non-etheless suggest that negative symptoms and social cognition are related, yet distinct constructs. Over time, deficits in social cognition could manifest behaviourally as negative symptoms (1). There are different models for a possible connection between social cognition and negative symptoms. On the one hand it is assumed that specific impairments in social cognition are linked to one or some negative symptoms but not others, which means there is a mechanistic heterogeneity. In support of this model, associations between only specific social cognition domains and negative symptoms have been reported (1). On the other hand, findings support broad impairments in basic sensory and cognitive processes (e.g., visual perception, motor output, processing speed, and implicit attention), which affect downstream processes. These are directly involved in social cognitive operations, which, in turn, result in the development of a range of negative symptoms and functional impairment (1). In studies looking at various factor models no correlations were found between the social cognitive factors and negative symptoms suggesting that social cognition and negative symptoms are largely separate constructs. They can be seen as relatively independent causes of dysfunction and disability and can be used to meaningfully classify non-acute patients (12, 28, 29). Furthermore, the entry level of negative symptoms was significantly associated with poor social functioning. Important predictive links have been found in the early course of schizophrenia, mostly indicating that higher negative symptom severity is associated with poor daily functioning and worse long-term outcomes (30–33). Given this, there is increasing interest in understanding to what amount negative symptoms reflect the expression of deficits in social cognition and the relationship between the two symptom domains (1). However, little is known about the dependence of these dimensions of illness and, whether individuals with schizophrenia can be meaningfully classified based on these dimensions and potentially differentially treated. Ruocco et al. (34), who compared emotion recognition deficits in individuals with schizophrenia, schizoaffective disorders, and bipolar disorders, discovered that compared to healthy controls, emotion recognition deficits among individuals increased progressively from bipolar disorder to schizoaffective disorder to schizophrenia. The researchers concluded that emotion recognition deficits are apparent at the first psychotic episode and prominent but different across psychotic disorders and relatively independent of mood state and antipsychotic treatment dosages (34–36). Conclusively illness-related characteristics like negative symptoms need further investigation (34–36). A priority of negative over positive symptoms in determining deficits in social cognition and functioning in chronic schizophrenia was concluded by many researchers (5, 37). To develop intervention approaches in the early phase of SSD, it is crucial to understand the longitudinal course of negative symptoms, especially in relationship to functioning (30).

There are, however, no sufficient treatment options for negative symptoms. The most often employed approaches as medication, psychotherapy, psychosocial interventions, and electroconvulsive therapy have only small effect sizes (38–40). A model of negative symptoms as the behavioral manifestation of altered social cognition has the potential to reveal areas of intact functioning, including domains of social cognition that remain unaffected by the illness, which novel treatments could rely upon to enhance the recovery of individuals with schizophrenia (1). The findings regarding social cognition in schizophrenia have treatment implications and the social processes that are aberrant in schizophrenia may each require their own specific therapeutic intervention. Training programmes that target facial emotion perception and mentalizing deficits have been validated in individuals with schizophrenia (2, 41, 42).

Some researchers, such as Schneider et al. (43), concluded that especially impairments in facial emotion perception were strongly associated with the severity of negative symptoms. Since then, several studies have investigated the connection between negative symptoms and emotion deficits, but findings on the relationship between emotion recognition and negative symptoms remain inconclusive (5, 15, 43–56). There are various explanations for these mixed results. Most of the studies did not differentiate subdomains of negative symptoms and only considered negative symptoms overall. Also, some studies did not include all six basic emotions in their experimental designs but presented the subjects with a smaller selection of emotional stimuli. Paul Ekman first defined the six basic emotions: anger, disgust, fear, happiness, sadness and surprise (15, 57). Furthermore, different operationalizations and instruments for negative symptoms could be a source of variation (58, 59). To better understand the link between specific negative symptoms and emotion recognition, this study aims to explore the relationship between recognizing the six basic emotions and seven distinct negative symptoms in individuals with SSD in an exploratory approach by using the Positive and Negative Syndrome Scale (PANSS) (60) and the Emotion Recognition Test of the Cambridge Neuropsychological Test Automated Battery (ERT-CANTAB) (61).

According to the current state of research, five key sub-domains of negative symptoms have been identified: (1) avolition, (2) anhedonia, (3) blunted affect (4) social withdrawal, and (5) alogia (9, 62–64). Negative symptoms can be assessed via various instruments [e.g., PANSS, Scale for the Assessment of Negative Symptoms (SANS) (65), Brief Negative Symptom Scale (BNSS) (66), Clinical Assessment Interview for Negative Symptoms (CAINS) (67), Negative Symptom Assessment (NSA) (68)]. So, it is necessary to consider the nature of each measurement instrument, especially with the aim of relating each symptom domain to the recognition of individual basic emotions. The domain of negative symptomatology is very complex encompassing primary and secondary symptoms as a result of medication side effects, depressive symptomatology and other causes (64). In SSD, studies confirmed moderate to large associations between negative symptoms and deficits in social cognition using the SANS (65) and/or the PANSS (1, 5, 12, 28, 30, 60, 69) as well as newer negative symptom scales like the BNSS (1, 5, 12, 66, 70). In many older studies, the negative syndrome scale of the classic PANSS was used to assess the individual domains of negative symptoms. However, according to recent factor analytic studies, it must be considered that the two symptom domains difficulty in abstract thinking (N5) and stereotyped thinking (N7) are now no longer regarded as negative symptoms but are assigned to the domain of cognitive symptoms (56) and difficulty in abstract thinking (N5) to the domain of disorganization, depending on the study design (71). This should therefore be considered when evaluating results using the PANSS. Nevertheless, in the context of an explorative approach of our study and for comparability with previous studies, especially older ones, we selected the PANSS as the primary rating tool for negative symptoms as a still frequently used third-party rating instrument in clinical studies (1, 5, 12, 28, 30, 60, 69). Depending on our results, a selection of the other measurement instruments should also be included in follow-up studies for an even better understanding of the relationships between negative symptoms and social cognition. We selected the Emotion Recognition Test of the CANTAB to measure the recognition of basic emotions. It contains all six basic emotions and is easy to use as a tablet-based measurement instrument in different settings (61). It is explicitly part of the Schizophrenia Test Battery of the CANTAB and several studies with patients with SSD have already been conducted with it. For example, Gica et al. (20) could show with the ERT-CANTAB, that emotion recognition deficits are long term stable features of schizophrenia and Kanchanatawan et al. (72) showed associations of anxiety and depressive symptoms in individuals with SSD with social cognition by using the ERT-CANTAB. Glenthøj et al. (22) could show impairments in social cognition with the ERT-CANTAB in ultra-high risk for psychosis individuals and that hereby emotion recognition latency but not accuracy relates to real life functioning in these individuals (21).

## Materials and Methods

### Sample and Procedure

The recruitment of participants was based on recommendations of a multiprofessional team. A sample of *N* = 66 individuals diagnosed with SSD was recruited at the in- (*n* = 36) and out-patients (*n* = 30) clinic of the Department for Psychiatry and Psychotherapy at the Charité – Universitätsmedizin Berlin, Germany (see Table 1). Of these, *n* = 54 were diagnosed with schizophrenia and *n* = 12 with a schizoaffective disorder. For inclusion, participants had to a) be aged between 18 to 65, b) meet diagnostic criteria for SSD (ICD-10: F2X.X) assessed by an attending psychiatrist and c) be able to give informed consent to participate in the study. Exclusion criteria encompassed (a) a score > 6 on any item of the positive scale of the Positive and Negative Syndrome Scale (PANSS) (60), which suggests an acute psychotic episode with severe psychotic symptoms, (b) acute suicidality assessed with the Clinical Decision Support System (CDSS) (item 8 > 1) (73), (c) current substance use other than nicotine or (d) the existence of neurological disorders or brain damages. For inpatients, the surveys were conducted within the first 8 weeks of treatment to avoid long hospitalization as a possible influencing factor on negative symptoms, respectively the exclusion criteria of an acute psychotic episode. The prescribed psychotropic medication and dosage was systematically recorded (see Table 2). Trained clinical staff assessed the seven different negative symptoms using the PANSS (60). The individuals‘ ability to recognize the six basic emotions happiness, sadness, fear, anger, surprise and disgust was measured using the Emotion Recognition Task (ERT) of the Cambridge Neuropsychological Test Automated Battery (CANTAB) (61) on an Apple® iPad Air using the software iPadOs 15.2. Basic demographic information of the study sample is summarized in Table 1.

**Table 1:** F2-Diagnoses and demographic data of the sample.

**Variable**	**Overall, *N* = 66**	**Schizoaffective disorder (F25), *n* = 12**	**Schizophrenia (F20), *n* = 54**
Sex			
Female	25 (38%)	8 (67%)	17 (31%)
Male	41 (62%)	4 (33%)	37 (69%)
Age			
N	66	12	54
Mean (SD)	41 (13)	50 (14)	40 (12)
Range	19, 69	26, 69	19, 67
Education			
Gymnasium (ISCED 3)	30 (46%)	6 (50%)	24 (45%)
Realschule (ISCED 2)	22 (34%)	5 (42%)	17 (32%)
Hauptschule (ISCED 2)	13 (20%)	1 (8.3%)	12 (23)
Missing	1	0	1

*N, sample size; SD, Standard Deviation; ISCED, International standard classification of education*.

**Table 2:** Overview of the antipsychotics taken during the study.

**Medication**	**Generation / potency**	**Mean (SD) (mg)**	**Administration**	**Number of patients**
Flupentixol	FG / HP	4.7 (3.1)	p.o.	3
Haloperidol	FG / HP	3	p.o.	1
Melperone	FG / LP	75	p.o.	1
Pipamperone	FG / LP	43.3 (29.4)	p.o.	6
Amisulpride	SG	553.9 (281)	p.o.	14
Aripiprazole	SG	13.4 (8.3)	p.o.	13
Aripiprazole	SG	300	i.m.	1
Cariprazine	SG	3 (0)	p.o.	2
Clozapine	SG	266 (239)	p.o.	22
Olanzapine	SG	19 (8.1)	p.o.	11
Paliperidone	SG	5.3 (4.5)	p.o.	5
Paliperidone	SG	112.5 (25)	i.m.	4
Quetiapine	SG	240 (176.1)	p.o.	10
Risperidone	SG	3.2 (1.2)	p.o.	12
Risperidone	SG	37.5	i.m.	1
Ziprasidone	SG	50 (42.4)	p.o.	2

*FG, First Generation; SG, Second generation; HP, High potency; LP, Low potency; mg, milligram; p.o., per os; i.m., intramuscular*.

### Measures

#### The Positive and Negative Syndrome Scale (PANSS)

The Positive and Negative Syndrome Scale (PANSS) is a clinical instrument specifically designed to measure symptoms associated with SSD on a 7-point scale (1 = absent, 2 = minimal, 3 = mild, 4 = moderate, 5 = moderate severe, 6 = severe and 7 = extreme). It encompasses three subscales; seven items to measure positive symptoms, seven items for negative symptoms and 16 on general symptomatology (60). Furthermore, the scale allows the calculation of a global score of overall symptom severity. These items are rated based on a comprehensive interview carried out by a trained clinician. The Negative Scale of the PANSS was used to assess the seven itemized negative symptoms: blunted affect (N1), emotional withdrawal (N2), poor rapport (N3), passive and apathetic social withdrawal (N4), difficulty in abstract thinking (N5), lack of spontaneity and flow of conversation (N6) and stereotyped thinking (N7). Regarding the psychometric properties of the Negative Scales of the PANSS, it can be said that the PANSS scores are normally distributed, and negative scales showed good interrater reliability. Negative syndromes also proved their factorial validity. The Negative Scale also held a high concurrent validity with the Scale for the Assessment of Negative Symptoms (SANS) (60, 65, 74).

#### Emotion Recognition Task (ERT)

The assessment of emotion recognition was carried out with the iPad version of the validated Cambridge Neuropsychological Test Automated Battery (CANTAB) in German language. The CANTAB is a computerized assessment developed from animal behavior paradigms and human neuropsychology, initially developed in the late 1980s (61). It has been widely used in many neurocognitive examinations of individuals with and without SSD (75). The largely non-verbal nature of the tests makes the CANTAB practical for use across multisite and multilingual clinical trials. The CANTAB schizophrenia test battery comprises computerized neuropsychological tests presented on a touchscreen system assessing eight cognitive domains most impaired in people affected by SSD. These captured cognitive domains were prioritized by the National Institute of Mental Health (NIMH) sponsored Measurement and Treatment Research to Improve Cognition in Schizophrenia (MATRICS) program (76). To assess social cognition within this study, we used the tablet-based Emotion Recognition Task (ERT), a facial emotion labeling task recently added to the battery, including testing for all six basic emotions: anger, disgust, fear, happiness, sadness, and surprise. Within this task participants are presented with computer-morphed faces expressing one specific emotion out of six basic emotions. Each face image is shown for 200 ms. The study participants are then asked to indicate the displayed emotion out of the six primary emotions or choose a neutral option. The task takes around 12 min to complete (61). Key outcome measures of the ERT are the overall median reaction time to select an emotion (ERTOMDRT), the total number of correct responses (total hits, ERTTH) and the unbiased hit rate for each of the six emotions: anger (ERTUHRA), disgust (ERTUHRD), fear (ERTUHRF), happiness (ERTUHRH), sadness (ERTUHRS) and surprise (ERTUHRSU).

### Ethical Approval

The current study was reviewed and approved by the Ethics Committee of Charité – Universitätsmedizin Berlin (EA4/225/19). All participants provided written informed consent to participate in this study.

### Statistical Analysis

The statistical analysis was carried out in R Studio Version 1.2.5033 running R version 4.0.4 and IBM SPSS Version 25. The following steps were performed for the statistical analyses in an exploratory approach of the study:

1. The Pearson's correlation coefficient was calculated between the seven negative symptoms and the accuracy and latency of recognition for each basic emotion.

2. Multiple linear regression models followed up the significant correlations to investigate the relationship while controlling for the covariates diagnoses, age, sex, and education. For covariate analysis, multiple linear regressions were run to examine the effect of each potential covariate on each CANTAB variable.

3. Each significant correlation between a negative symptom and a CANTAB variable was tested as a linear regression model by including the significant covariates in the regression models.

4. Bonferroni corrections for multiple testing were performed despite a not sufficient sample size for statistical completeness. The Bonferroni adjusted *p*-value is reported as p_*Bonf*_ respectively (77). Power and sample size calculations followed.

## Results

**Figure 1:**
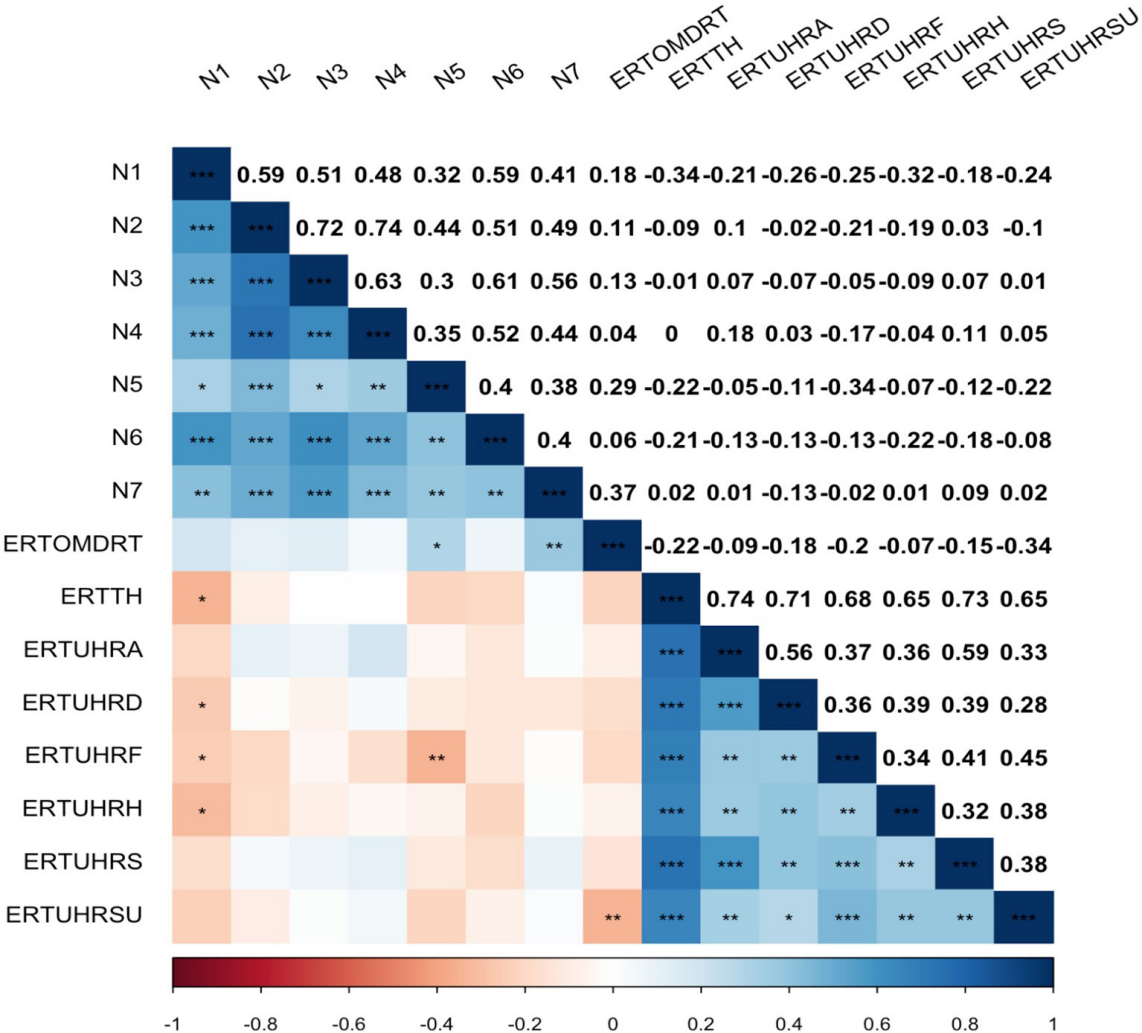
Correlation Matrix of Positive and Negative Syndrome Scale (PANSS) Negative Scale and Emotion Recognition Test (ERT) key outcomes in form of a heat map with Pearson correlation coefficient and significance level with unadjusted *p*-values (***p < 0.001, **p < 0.01, *p < 0.05); N1, blunted affect; N2, emotional withdrawal; N3, poor rapport; N4, passive and apathetic social withdrawal; N5, difficulty in abstract thinking; N6, lack of spontaneity and flow of conversation; N7, stereotyped thinking; ERTOMDRT, the overall median reaction time to select an emotion; ERTTH, the total number of correct responses; ERTUHRA, the unbiased hit rate for the emotion anger; ERTUHRD, the unbiased hit rate for the emotion disgust; ERTUHRF, the unbiased hit rate for the emotion fear; ERTUHRH, the unbiased hit rate for the emotion happiness; ERTUHRS, the unbiased hit rate for the emotion sadness; ERTUHRSU, the unbiased hit rate for the emotion surprise.

In Figure 1, a correlational matrix with an overview of the findings that emerged after data collection is presented. Positive correlations within the respective scales can also be found in the literature (60, 78–80). There was a significant negative correlation between blunted affect (N1) and the total number of correct responses in emotion selection (*r* = −0.291, *p* < 0.05), the recognition of happiness (*r* = −0.034, *p* = < 0.05), fear (*r* = −0.246, *p* < 0.05) and disgust (*r* = −0.253, *p* < 0.05). A significant negative correlation of difficulties in abstract thinking (N5) with fear recognition (*r* = −0.337, *p* < 0.05) was found. There was also a significant positive correlation of the response latency in emotion recognition with difficulties in abstract thinking (N5) (*r* = 0.289, *p* < 0.05) and stereotyped thinking (N7) (*r* = 0.349, *p* < 0.05). There were no significant correlations between the negative symptom domains emotional withdrawal (N2), poor rapport (N3), passive and apathetic social withdrawal (N4), and lack of spontaneity and flow of conversation (N6) and the assessed CANTAB variables among those with SSD. In the next step, linear regressions were run to examine a significant effect of the covariates age, education, sex and diagnoses on each CANTAB variable.

When controlling for age and education, a multiple linear regression analysis was conducted to assess whether blunted affect (N1) significantly predicted the total number of correct responses in emotion selection. Hereby blunted affect (N1) significantly predicted the total number of correct responses in emotion selection [*B* = −0.919, *t* = −2.199, *p* = 0.032]. Blunted affect (N1) could also be identified as a significant predictor of the accurate recognition of happiness when controlling for the covariates [*B* = −0.241, *t* = −2.113, *p* = 0.039]. Likewise, the negative symptom domain of difficulties in abstract thinking (N5) was identified as a significant predictor of fear recognition in the regression analyses [*B* = −0.037, *t* = −2.044, *p* = 0.045] when controlling for education. A linear regression analysis was conducted to assess whether difficulties in abstract thinking (N5) and stereotyped thinking (N7) predicted the response latency when controlling for age. In this model stereotyped thinking (N7) significantly predicted the response latency [*B* = 195.921, *t* = 2.400, *p* = 0.019], whereas the negative symptom difficulties in abstract thinking (N5) was not a significant predictor in this model [*B* = 117.474, *t* = 1.535, *p* = 0.130]. Linear regression analysis models showed that that blunted affect (N1) was not a significant predictor for recognizing either fear [*B* = −0.023, *t* = −1.607, *p* = 0.113] or disgust [*B* = −0.027, *t* = −1.699, *p* = 0.094], while controlling for the significant covariate education. For an overview of the above significant regression analyses, please refer to Table 3. The complete respective regression tables are provided in the Supplementary Material.

**Table 3:** Regression analysis predicting the ERT items resulting from the correlation analyses.

**Independent Variable**	**Unstandardized coefficient**		**Standardized coefficient**	***t***	***p***	***p**_***Bonf***_*	**Dependent variable**
	**B**	**SE**	**Beta**				
N1	−0.919	0.418	−0.241	−2.199	0.032	1.000	ERTTH
N1	−0.034	0.016	−0.241	−2.113	0.039	1.000	ERTUHRH
N5	−0.037	0.018	−0.270	−2.044	0.045	1.000	ERTUHRF
N7	195.921	81.634	0.287	2.400	0.019	1.000	ERTOMDRT

*Constant = 555.393, F(3,62) = 6,402, p = 0.001, R^2^ = 0.200; p_Bonf_, Bonferroni adjusted p-value; N1, blunted affect; N5, difficulty in abstract thinking; N7, stereotyped thinking; ERTTH, the total number of correct responses; ERTUHRH, the unbiased hit rate for the emotion happiness; ERTUHRF, the unbiased hit rate for the emotion fear; ERTOMDRT, the overall median reaction time to select an emotion*.

The demonstrated nominal significances did not withstand correction for 111 multiple tests. After Bonferroni correction, none of the mentioned correlation and regression analyses between PANSS and the ERT items, respectively, reached statistical significance (see Supplementary Material). With a power of 0.8, an expected small effect of *f2* = 0.054 of the PANSS negative scales on emotion recognition based on preliminary studies (81), and a *p*-value of 0.00045 corrected for 111 multiple tests, a sample size of 446 subjects would be necessary to detect a significant effect. According to the guidelines of Cohen (82) *f2* ≥ 0.02, *f2* ≥ 0.15, and *f2* ≥ 0.35 represent small, medium, and large effect sizes, respectively.

## Discussion

### Summary of the Main Results

In the present study, blunted affect (N1) emerged as the negative symptom most strongly predicting the total number of correct responses, especially happiness recognition. First, a significant correlation was shown between blunted affect (N1) and happiness, fear and disgust. After controlling for the significant covariates, blunted affect (N1) only significantly predicted the recognition of happiness. We also found a prediction of difficulties in abstract thinking (N5) for recognizing the basic emotion of fear. Additionally, a significant correlation between difficulties in abstract thinking (N5) and response latency and a significant prediction of stereotyped thinking (N7) for the response latency in emotion recognition was found. However, results must be weighed against the methodological limitations of the study.

### Prediction for Response Latency in Emotion Recognition

With reference to the results regarding response latency and the association with stereotyped thinking (N7) and difficulty in abstract thinking (N5), some authors assign stereotyped thinking, difficulties in abstract thinking alongside conceptual disorganization, disorientation, and poor attention rather to a cognitive symptom dimension than to the negative symptom dimension (56). This could explain the prediction for the response latency, where cognitive processing of impressions may play a role. Glenthøj et al. (21), also using the CANTAB for emotion recognition, reported that latency in emotion recognition, but not accuracy, related to real-life functioning for individuals at ultra-high risk for psychosis (21). More studies have been conducted on the relationship between cognitive capabilities and the ability to recognize emotions in individuals with SSD, but again with inconclusive results. According to Turetsky et al. (83), emotion recognition deficits could be assumed to be secondary to problems in a structural encoding of faces, and a specific signal recognition could play a role that precedes emotion recognition. Additionally, several studies reported a clear association between neuropsychological functions and emotion recognition (43, 56, 84, 85).

### Prediction for the Correct Recognition of the Six Emotions

Blunted affect (N1) significantly predicted the recognition of happiness. The current study results have been confirmed in the literature, while other studies reported inconclusive results and need further classification (15). On the one hand some authors like Turetsky et al. (83) showed that recognizing happy faces correlated with the severity of negative symptoms, especially in individuals with SSD. On the other hand, ratings in the blunted effect's subdomain uniquely predicted performance on the emotion processing tasks. They were associated with better speed and accuracy than other negative symptom domains (8). Blunted affect being more evident in men than women is in line with the findings of Kohler et al. (84), showing a poorer performance of men in emotion recognition. Several studies examined the relationship between impaired emotion perception with different symptom rating scales, e.g., the Brief Psychiatric Rating Scale (BPRS) (86), the Scale for the Assessment of the Positive Symptoms (SAPS) (87), the SANS (65), and the PANSS (60). Their impact and differences in the sample recruitment may have contributed to the inconsistencies reported in the literature (44) and should be further pursued. Since emotional impairments are connected to negative symptoms such as blunted affect and influence the course of illness from the onset on (8, 23, 55, 88), it would be important to focus on emotion recognition and early detection, especially regarding young individuals with SSD.

In some studies, the impaired recognition of positive emotions was found to be a prominent deficit in individuals with schizophrenia (34, 89) and significant group differences between individuals with SSD and healthy individuals were only limited to positive emotions like happiness (55, 56, 84). Tsoi et al. (89) showed that recognizing happy faces was more impaired than recognizing sad or fearful faces in a sample with individuals with schizophrenia. It could be shown that the perception of happy and sad emotions also relates differently to significant illness parameters such as age, intelligence quotient (IQ), cumulated time in hospital and negative symptoms (7, 48, 90). This supports the idea of the existence of an emotion-specific deficit in the perception of emotions in individuals with schizophrenia and of at least two separate neurobiological pathways for processing positive and negative emotions (18, 48, 89, 91–95). The inconsistencies of the findings on differential abilities to recognize positive vs. negative affect states could also be due to methodological, stimuli and sampling differences or other as yet unknown variables (18, 56).

### Implications for Future Research and Future Practice

Despite the needs and hopes for therapeutic interventions in the field of emotion recognition for individuals with SSD, many aspects in this field of research must be further clarified in future studies. Finally, a study design with a larger sample to follow up on the investigated predictors within the framework of our exploratory study would be helpful. This study initially pursued an exploratory approach to shed more light on the associations between negative symptoms and emotion recognition. Each significant correlation between a negative symptom and a CANTAB variable was tested as a linear regression model by including the significant covariates in the regression models. The nominal significances of our exploratory study did not withstand Bonferroni correction for multiple tests, which was performed despite a not sufficient sample size for statistical completeness in the exploratory approach. Nevertheless, this explorative approach yielded results that can be built upon in follow-up studies with larger sample sizes, additional measurement instruments, and covariates. The correlation matrix (see Figure 1) shows significant within-correlations of all negative symptoms, some of which remain significant even after Bonferroni correction (see Supplementary Material). Therefore, the question arises whether the individual negative symptoms can even independently predict the ERT variables. Also, a larger sample could be used to calculate additional regression models in which all negative symptoms are included in future studies.

For covariate analysis, multiple linear regressions were run to examine the effect of each potential covariate on each CANTAB variable in our sample. Due to the initially exploratory approach of our study with a small sample size, the analyses were initially performed with a limited and selected number of covariates to control for. The variables that were expected to be most informative in the context of the study were diagnosis, gender, age, and education, as these include biological and cognitive components. The antipsychotic medication of the patients was systematically recorded and can be seen in Table 2. Due to the exploratory approach of our study with a small sample and the heterogeneity in medication, we did not statistically control for these. Since it could be shown that emotion recognition is relatively independent of the influence of antipsychotics (34–36) and most antipsychotics of our sample belonged to second generation antipsychotics with only a minor influence on negative symptoms and cognitive functions (96), medication was considered as a negligible influence factor concerning the sample size. However, the heterogeneity of the collected medication in our sample reflects the treatment of patients with SSD for generalizability of the results. To allow generalizability of our study sample in this exploratory approach, we wanted to recruit as heterogeneous a cohort as possible, which also reflects a realistic representation of individuals with SSD in the inpatient and outpatient setting. In a large-scale follow-up study based on our results, further covariates like neurocognition, positive symptoms, medication and duration of illness and hospitalization should be therefore considered. A distinction between individuals with schizophrenia, schizoaffective disorders, and other diagnoses of SSD especially according to the ICD-11 classification regarding emotion recognition would be also of interest for future research in a larger sample. To reach the potential of personalized medicine, associations with the severity of predominant symptom manifestations should also be further illuminated in the framework of larger samples (34).

Additionally different measurements for negative symptoms are needed to ensure comparability. We initially chose the PANSS as the primary rating tool for negative symptoms within the framework of an explorative approach for the purpose of comparability with previous studies, especially older studies and due to inconsistencies in literature. The PANSS is a still frequently used external rating instrument in clinical studies. However, the limitations of the PANSS with respect to the nature of the negative symptom domains must certainly be considered in the interpretation of the results. Thus, as already mentioned, in factor analyses difficulty in abstract thinking (N5) and stereotyped thinking (N7) are attributed to the cognitive symptom domain (56) and difficulty in abstract thinking (N5) can be also accounted to the domain of disorganization (71). New generation scales like BNSS, CAINS and NSA should be included in follow-up studies to assess negative symptoms for an even broader understanding of their relationships with social cognition. Since most of the scales evaluate the behavioral side of negative symptoms, while the emotion recognition tasks assess the subjective experience, the new Self-evaluation of Negative Symptoms (SNS) (97) as a self-rating instrument would be an option for an additional perspective. Another alternative measure to be included is the recently introduced PANSS Autism Severity Score (PAUSS) (98, 99). The PAUSS is a score obtained from – essentially – the negative scale of the PANSS aimed to resemble autism spectrum disorder features in people with schizophrenia. It is a new construct with respect to autism conceptualized by classical continental psychopathology, as in the Autism Rating Scale (ARS) (100) and has already been studied in relation to social cognition (98).

Another reason for using the PANSS for our study was the ERT-CANTAB as an already relatively new measurement instrument to be chosen to capture emotion recognition. Hereby an advantage is the feasibility on a tablet and the thereby simple manageability. In addition, in studies by Glenthøj et al. (21) ERT-CANTAB was used in UHR for psychosis, so that in follow-up studies these individuals could be also included regarding the fact that impairments in emotion recognition and negative symptoms can already be determined before the onset of other symptoms (10, 11). However, different survey instruments for emotion recognition should be considered in follow-up studies to obtain a better insight independent of the measurement instrument. A comparison should therefore be made with likewise established measurement instruments for emotion recognition such as the Facial Emotion Identification Test (FEIT) (95), the Bell-Lysaker Emotion Recognition Task (BLERT) (101) or The Awareness of Social Inference Test (TASIT) (102). A systematic comparison of the measurement instruments would be of great benefit for clarifying the inconclusive results in literature. Research should be also expanded on all modalities of emotion recognition impaired in individuals with SSD (7, 15), including visual, verbal, and auditory channels (15). In addition, a longitudinal study and a study design for causality statements would be useful.

Consequently, impairments identified in the subdomains of negative symptoms and emotion recognition should be considered for early detection and individualized treatment as one of the research goals in social cognition in individuals with SSD, considering the enormous social burden. For example, specific interaction and emotion recognition training for a subgroup of individuals with specific profiles of negative symptoms, for instance in analogy to training programs for individuals with autism spectrum disorders (103, 104), could be developed to improve the recovery of individuals with SSD. Another treatment option that should be examined more closely in this area is the use of the neuropeptide oxytocin. In some studies, nasal oxytocin administration could improve the social performance of individuals with SSD (105).

### Strength and Limitations

Concerning the size and selection of our exploratory study sample, larger diverse samples are needed to allow more precise analyses regarding the different variables. Our sample size calculations showed a sample size of 446 subjects required to detect a significant effect after correction for multiple testing. As could be expected, the nominal significances of our exploratory study did not withstand correction for multiple tests. Therefore, follow-up studies are necessary and recruitment for the present study is continued. Our research focused on negative symptoms, so we did not include positive symptom scores or total PANSS scores as well as further covariates like neurocognition. Only the CANTAB was used as a measurement instrument for emotion recognition. No comparison was made with other measurement instruments so that individual characteristics of the battery could have influenced the results. Additionally, different measurements for negative symptoms are needed to avoid bias and ensure comparability. Our results were collected in a cross-sectional design, so statements are limited to significant predictors in emotion recognition.

## Conclusion

Conclusively in our exploratory study individuals with SSD and high scores of the negative symptoms blunted affect (N1), difficulty in abstract thinking (N5) and stereotyped thinking (N7) showed impairments in recognizing basic emotions and, here, particularly happiness. However, results must be weighed against the methodological limitations of the study and follow-up studies are necessary.

## Data Availability Statement

The raw data supporting the conclusions of this article will be made available by the authors, without undue reservation.

## Ethics Statement

The studies involving human participants were reviewed and approved by Charité's Ethics Committee. The patients/participants provided their written informed consent to participate in this study.

## Author Contributions

MZ, KB, NB, IH, AB, and JK designed and executed the study and conducted the data analyses. MZ wrote the article. TT, SR, EH, and MB collaborated with the design and editing of the manuscript. All authors contributed to the article and approved the submitted version.

## Funding

MZ is a participant in the BIH-Charité Junior Clinician Scientist Program funded by the Charité – Universitätsmedizin Berlin and the Berlin Institute of Health (BIH). In addition, the study was funded through the internal performance-based funding (LoM) of our research group at Charité – Universitätsmedizin Berlin.

## Conflict of Interest

The authors declare that the research was conducted in the absence of any commercial or financial relationships that could be construed as a potential conflict of interest.

## Publisher's Note

All claims expressed in this article are solely those of the authors and do not necessarily represent those of their affiliated organizations, or those of the publisher, the editors and the reviewers. Any product that may be evaluated in this article, or claim that may be made by its manufacturer, is not guaranteed or endorsed by the publisher.
