# Supplementary material for: The Relationship Between the Recognition of Basic Emotions and Negative Symptoms in Individuals With Schizophrenia Spectrum Disorders – An Exploratory Study
Source: Front Psychiatry. 2022 Apr 27;13:865226. doi: 10.3389/fpsyt.2022.865226 (PMC9091587; doi:10.3389/fpsyt.2022.865226)
Supplement: Supplementary file 1 [file Data_Sheet_1.docx]

Supplementary Material

# Supplementary Tables

**Supplementary Table 1.**

*Regression analysis predicting the total number of correct responses (ERTTH).*

| **Independent Variable** | **Unstandardized coefficient** | | **Standardized coefficient** | ***t*** | ***p*** | ***p_Bonf_*** |
| --- | --- | --- | --- | --- | --- | --- |
|  | **B** | **SE** | **Beta** |  |  |  |
| N1 | -.919 | .418 | -.241 | -2.199 | .032 | 1.000 |
| Age | -.165 | .049 | -.364 | -3.377 | .001 |  |
| Education | 1.984 | .812 | .268 | 2.443 | .017 |  |

*Note.* Constant = 32.704, *F*(3,61) = 8,475, *p* < .001, *R^2^* = .259, *p_Bonf_* = Bonferroni adjusted *p*-value, N1 *=* blunted affect.

**Supplementary Table 2.**

*Regression analysis predicting the hit rate for the emotion happiness (ERTUHRH).*

| **Independent Variable** | **Unstandardized coefficient** | | **Standardized coefficient** | ***t*** | ***p*** | ***p_Bonf_*** |
| --- | --- | --- | --- | --- | --- | --- |
|  | **B** | **SE** | **Beta** |  |  |  |
| N1 | -.034 | .016 | -.241 | -2.113 | .039 | 1.000 |
| Age | -.005 | .002 | -.303 | -2.689 | .009 |  |
| Education | .063 | .031 | .232 | 2.026 | .047 |  |

*Note.* Constant = .720, *F*(3,61) = 6,08, *p* = 0,001, *R^2^* = .192, *p_Bonf_* = Bonferroni adjusted *p*-value, N1 *=* blunted affect.

**Supplementary Table 3.**

*Regression analysis predicting the hit rate for the emotion fear (ERTUHRF).*

| **Independent Variable** | **Unstandardized coefficient** | | **Standardized coefficient** | ***t*** | ***p*** | ***p_Bonf_*** |
| --- | --- | --- | --- | --- | --- | --- |
|  | **B** | **SE** | **Beta** |  |  |  |
| N5 | -.037 | .018 | -.270 | -2.044 | .045 | 1.000 |
| Education | .032 | .029 | .143 | 1.077 | .286 |  |

*Note.* Constant = .238*, F*(2,61) = 4,410, p = .016, *R^2^* = .098, *p_Bonf_* = Bonferroni adjusted *p*-value, N5 = difficulty in abstract thinking.

**Supplementary Table 4.**

*Regression analysis predicting the overall median reaction time (ERTOMDRT).*

| **Independent Variable** | **Unstandardized coefficient** | | **Standardized coefficient** | | ***t*** | ***p*** | ***p_Bonf_*** |
| --- | --- | --- | --- | --- | --- | --- | --- |
|  | **B** | **SE** | | **Beta** |  |  |  |
| N5 | 117.474 | 76.511 | | .184 | 1.535 | .130 | 1.000 |
| N7 | 195.921 | 81.634 | | .287 | 2.400 | .019 |  |
| Age | 19.043 | 7.235 | | .292 | 2.632 | .011 |  |

*Note.* Constant = 555.393, *F*(3,62) = 6,402, *p* = .001, *R^2^* = .200, *p_Bonf_* = Bonferroni adjusted *p*-value, N5 = difficulty in abstract thinking, N7 = stereotyped thinking.

**Supplementary Table 5.**

*Adjusted p-values for correlations and linear regression.*

| **Variable 1** | **Variable 2** | **Analysis** | ***p*** | ***p_Bonf_*** | **Significance** |
| --- | --- | --- | --- | --- | --- |
| N1 | N2 | correlation | 0.000 | 0.000 | **** |
| N1 | N3 | correlation | 0.000 | 0.001 | *** |
| N1 | N4 | correlation | 0.000 | 0.003 | ** |
| N1 | N5 | correlation | 0.011 | 1.000 |  |
| N1 | N6 | Correlation | 0.000 | 0.000 | **** |
| N1 | N7 | correlation | 0.002 | 0.187 |  |
| N1 | ERTTH | correlation | 0.018 | 1.000 |  |
| N1 | ERTOMDRT | correlation | 0.118 | 1.000 |  |
| N1 | ERTUHRA | correlation | 0.126 | 1.000 |  |
| N1 | ERTUHRD | correlation | 0.040 | 1.000 |  |
| N1 | ERTUHRF | correlation | 0.048 | 1.000 |  |
| N1 | ERTUHRH | correlation | 0.021 | 1.000 |  |
| N1 | ERTUHRS | correlation | 0.221 | 1.000 |  |
| N1 | ERTUHRSU | correlation | 0.110 | 1.000 |  |
| N2 | N3 | correlation | 0.000 | 0.000 | **** |
| N2 | N4 | correlation | 0.000 | 0.000 | **** |
| N2 | N5 | correlation | 0.000 | 0.034 |  |
| N2 | N6 | correlation | 0.000 | 0.001 | *** |
| N2 | N7 | correlation | 0.000 | 0.012 |  |
| N2 | ERTTH | correlation | 0.655 | 1.000 |  |
| N2 | ERTOMDRT | correlation | 0.349 | 1.000 |  |
| N2 | ERTUHRA | correlation | 0.349 | 1.000 |  |
| N2 | ERTUHRD | correlation | 0.903 | 1.000 |  |
| N2 | ERTUHRF | correlation | 0.100 | 1.000 |  |
| N2 | ERTUHRH | correlation | 0.225 | 1.000 |  |
| N2 | ERTUHRS | correlation | 0.668 | 1.000 |  |
| N2 | ERTUHRSU | correlation | 0.600 | 1.000 |  |
| N3 | N4 | correlation | 0.000 | 0.000 | **** |
| N3 | N5 | correlation | 0.019 | 1.000 |  |
| N3 | N6 | correlation | 0.000 | 0.000 | **** |
| N3 | N7 | correlation | 0.000 | 0.001 | ** |
| N3 | ERTTH | correlation | 0.878 | 1.000 |  |
| N3 | ERTOMDRT | correlation | 0.255 | 1.000 |  |
| N3 | ERTUHRA | correlation | 0.481 | 1.000 |  |
| N3 | ERTUHRD | correlation | 0.547 | 1.000 |  |
| N3 | ERTUHRF | correlation | 0.719 | 1.000 |  |
| N3 | ERTUHRH | correlation | 0.618 | 1.000 |  |
| N3 | ERTUHRS | correlation | 0.484 | 1.000 |  |
| N3 | ERTUHRSU | correlation | 0.749 | 1.000 |  |
| N4 | N5 | correlation | 0.005 | 0.576 |  |
| N4 | N6 | correlation | 0.000 | 0.001 | *** |
| N4 | N7 | correlation | 0.000 | 0.048 |  |
| N4 | ERTTH | correlation | 0.784 | 1.000 |  |
| N4 | ERTOMDRT | correlation | 0.661 | 1.000 |  |
| N4 | ERTUHRA | correlation | 0.121 | 1.000 |  |
| N4 | ERTUHRD | correlation | 0.773 | 1.000 |  |
| N4 | ERTUHRF | correlation | 0.170 | 1.000 |  |
| N4 | ERTUHRH | correlation | 0.903 | 1.000 |  |
| N4 | ERTUHRS | correlation | 0.310 | 1.000 |  |
| N4 | ERTUHRSU | correlation | 0.557 | 1.000 |  |
| N5 | N6 | correlation | 0.001 | 0.123 |  |
| N5 | N7 | correlation | 0.002 | 0.210 |  |
| N5 | ERTTH | correlation | 0.076 | 1.000 |  |
| N5 | ERTOMDRT | correlation | 0.018 | 1.000 |  |
| N5 | ERTUHRA | correlation | 0.698 | 1.000 |  |
| N5 | ERTUHRD | correlation | 0.362 | 1.000 |  |
| N5 | ERTUHRF | correlation | 0.006 | 0.680 |  |
| N5 | ERTUHRH | correlation | 0.593 | 1.000 |  |
| N5 | ERTUHRS | correlation | 0.347 | 1.000 |  |
| N5 | ERTUHRSU | correlation | 0.080 | 1.000 |  |
| N6 | N7 | correlation | 0.002 | 0.196 |  |
| N6 | ERTTH | correlation | 0.152 | 1.000 |  |
| N6 | ERTOMDRT | correlation | 0.581 | 1.000 |  |
| N6 | ERTUHRA | correlation | 0.371 | 1.000 |  |
| N6 | ERTUHRD | correlation | 0.319 | 1.000 |  |
| N6 | ERTUHRF | correlation | 0.295 | 1.000 |  |
| N6 | ERTUHRH | correlation | 0.127 | 1.000 |  |
| N6 | ERTUHRS | correlation | 0.202 | 1.000 |  |
| N6 | ERTUHRSU | correlation | 0.658 | 1.000 |  |
| N7 | ERTTH | correlation | 0.949 | 1.000 |  |
| N7 | ERTOMDRT | correlation | 0.004 | 0.449 |  |
| N7 | ERTUHRA | correlation | 0.987 | 1.000 |  |
| N7 | ERTUHRD | correlation | 0.400 | 1.000 |  |
| N7 | ERTUHRF | correlation | 0.845 | 1.000 |  |
| N7 | ERTUHRH | correlation | 0.970 | 1.000 |  |
| N7 | ERTUHRS | correlation | 0.565 | 1.000 |  |
| N7 | ERTUHRSU | correlation | 0.978 | 1.000 |  |
| ERTTH | ERTOMDRT | correlation | 0.099 | 1.000 |  |
| ERTTH | ERTUHRA | correlation | 0.000 | 0.000 | **** |
| ERTTH | ERTUHRD | correlation | 0.000 | 0.000 | **** |
| ERTTH | ERTUHRF | correlation | 0.000 | 0.000 | **** |
| ERTTH | ERTUHRH | correlation | 0.000 | 0.000 | **** |
| ERTTH | ERTUHRS | correlation | 0.000 | 0.000 | **** |
| ERTTH | ERTUHRSU | correlation | 0.000 | 0.000 | **** |
| ERTOMDRT | ERTUHRA | correlation | 0.503 | 1.000 |  |
| ERTOMDRT | ERTUHRD | correlation | 0.138 | 1.000 |  |
| ERTOMDRT | ERTUHRF | correlation | 0.114 | 1.000 |  |
| ERTOMDRT | ERTUHRH | correlation | 0.617 | 1.000 |  |
| ERTOMDRT | ERTUHRS | correlation | 0.252 | 1.000 |  |
| ERTOMDRT | ERTUHRSU | correlation | 0.008 | 0.865 |  |
| ERTUHRA | ERTUHRD | correlation | 0.000 | 0.000 | *** |
| ERTUHRA | ERTUHRF | correlation | 0.003 | 0.289 |  |
| ERTUHRA | ERTUHRH | correlation | 0.003 | 0.283 |  |
| ERTUHRA | ERTUHRS | correlation | 0.000 | 0.000 | **** |
| ERTUHRA | ERTUHRSU | correlation | 0.005 | 0.586 |  |
| ERTUHRD | ERTUHRF | correlation | 0.004 | 0.391 |  |
| ERTUHRD | ERTUHRH | correlation | 0.001 | 0.137 |  |
| ERTUHRD | ERTUHRS | correlation | 0.002 | 0.187 |  |
| ERTUHRD | ERTUHRSU | correlation | 0.025 | 1.000 |  |
| ERTUHRF | ERTUHRH | correlation | 0.005 | 0.556 |  |
| ERTUHRF | ERTUHRS | correlation | 0.001 | 0.075 |  |
| ERTUHRF | ERTUHRSU | correlation | 0.000 | 0.020 |  |
| ERTUHRH | ERTUHRS | correlation | 0.008 | 0.854 |  |
| ERTUHRH | ERTUHRSU | correlation | 0.001 | 0.120 |  |
| ERTUHRS | ERTUHRSU | correlation | 0.001 | 0.117 |  |
| N1 | ERTTH | regression | 0.049 | 1.000 |  |
| N1 | ERTUHRH | regression | 0.066 | 1.000 |  |
| N5 | ERTUHRF | regression | 0.043 | 1.000 |  |
| N5 | ERTOMDRT | regression | 0.165 | 1.000 |  |
| N7 | ERTOMDRT | regression | 0.062 | 1.000 |  |

*Note.* *p_Bonf_* = Bonferroni adjusted *p*-value, N1 *=* blunted affect, N2 = emotional withdrawal, N3 = poor rapport, N4 = passive and apathetic social withdrawal, N5 = difficulty in abstract thinking, N6 = lack of spontaneity and flow of conversation, N7 = stereotyped thinking, ERTOMDRT = the overall median reaction time to select an emotion, ERTTH = the total number of correct responses, ERTUHRA = the unbiased hit rate for the emotion anger, ERTUHRD = the unbiased hit rate for the emotion disgust, ERTUHRF = the unbiased hit rate for the emotion fear, ERTUHRH = the unbiased hit rate for the emotion happiness, ERTUHRS = the unbiased hit rate for the emotion sadness, ERTUHRSU = the unbiased hit rate for the emotion surprise.
